# GABAergic Influences on Medulloblastoma

**DOI:** 10.3390/brainsci15070746

**Published:** 2025-07-11

**Authors:** Viviane Aline Buffon, Jurandir M. Ribas Filho, Osvaldo Malafaia, Isadora D. Tassinari, Rafael Roesler, Gustavo R. Isolan

**Affiliations:** 1 Graduate Program in Principles of Surgery, Mackenzie Evangelical University, Curitiba 80730-000, Brazil; 2 Laboratory of Neurobiology and Metabolism (NeuroMet), Department of Physiology, Institute for Basic Health Sciences, Federal University of Rio Grande do Sul, Porto Alegre 90035-003, Brazil; 3 Graduate Program in Physiology, Institute for Basic Health Sciences, Federal University of Rio Grande do Sul, Porto Alegre 90035-003, Brazil; 4 Department of Pharmacology, Institute for Basic Health Sciences, Federal University of Rio Grande do Sul, Porto Alegre 90035-003, Brazil; 5 Cancer and Neurobiology Laboratory, Experimental Research Center, Clinical Hospital (CPE-HCPA), Federal University of Rio Grande do Sul, Porto Alegre 90035-003, Brazil; 6 National Science and Technology Institute for Children’s Cancer Biology and Pediatric Oncology–INCT BioOncoPed, Porto Alegre 90035-003, Brazil; 7 The Center for Advanced Neurology and Neurosurgery (CEANNE), Porto Alegre 90560-010, Brazil

**Keywords:** γ-aminobutyric acid, GABA_A_ receptor, *GABRA5*, GABA_A_ α5 subunit, medulloblastoma, pediatric brain cancer

## Abstract

Medulloblastoma (MB) is the most common malignant brain tumor in children and typically arises in the cerebellum, likely due to disruptions in neuronal precursor development. The primary inhibitory neurotransmitter in the central nervous system (CNS), γ-aminobutyric acid (GABA), exerts its effects through GABA_A_, GABA_B_, and GABA_C_ receptors. GABA receptor activity regulates the development and function of cerebellar neurons, including glutamatergic cerebellar granule cells (CGCs). Beyond the nervous system, GABA is also a common metabolite in non-neuronal cell types. An increasing body of evidence indicates that GABA can influence cell proliferation, differentiation, and migration in several types of adult solid tumors, including brain cancers. GABA and GABA_A_ receptor agonists can impair the viability and survival of MB cells, primarily acting on GABA_A_ receptors containing the α5 subunit. A marked expression of the gene encoding the α5 subunit is found across all MB tumor molecular subgroups, particularly Group 3 MB, which has a poor prognosis. Importantly, high levels of the γ-aminobutyric acid type A receptor subunit α5 *(GABRA5*) gene are associated with shorter patient overall survival in Group 3 and Group 4 MB. In contrast, high γ-aminobutyric acid type A receptor subunit β1 (*GABRB1*) gene expression is related to longer survival in all MB subgroups. The GABAergic system may, therefore, regulate MB cell function and tumor progression and influence patient prognosis, and is worthy of further investigation as a biomarker and therapeutic target in MB.

## 1. Introduction

Brain cancers are the most common solid tumors in children and the leading cause of cancer-related mortality in this population. Medulloblastoma (MB), the most common malignant pediatric brain tumor, is an embryonal cancer arising in the cerebellum that illustrates a tumor type that originates from failures in neurodevelopment. Despite significant improvements in multimodal treatments consisting of surgery, radiotherapy, and chemotherapy, approximately one-third of patients experience relapse, often with poor outcomes. Survivors frequently endure long-term neurological, cognitive, and endocrinological side effects due to the aggressive nature of therapy [1].

The major inhibitory neurotransmitter in the central nervous system (CNS) is γ-aminobutyric acid (GABA), which acts by binding to different types of GABA receptors. Rapid neuronal inhibition is mediated by activation of the GABA_A_ type of receptor, which forms a ligand-gated chloride (Cl^−^) ion channel. In most adult neurons, increased GABA-induced Cl^−^ influx results in cell membrane hyperpolarization and, ultimately, neuronal inhibition [2,3]. In addition to mediating fast neuronal inhibition in the adult brain, GABA and its receptors regulate the proliferation and differentiation of neural stem cells and neuronal progenitors [4,5] and regulate cerebellar development [6,7]. In addition, changes in GABAergic transmission may be involved in neurodevelopmental disorders, including autism, Rett syndrome, Down syndrome, neurofibromatosis type I, fragile X syndrome, and schizophrenia [8]. The pharmacological modulation of GABAergic activity mediated by GABA_A_ receptors can influence MB cells, and current evidence shows associations between the gene expression of different GABA_A_ receptor subunits and survival of pediatric MB patients [9,10,11]. Here, we review the relationship between GABAergic neurotransmission and MB growth, as well as the possible impact of GABA receptor subunit expression on patient prognosis.

## 2. Origins of MB

Major advancements in genomic, epigenomic, and transcriptomic research have revealed that MB is a highly heterogeneous disease, showing substantial plasticity of tumor cell populations, many of which mirror transient fetal cerebellar transcriptional programs [12,13,14,15,16,17]. MB tumors are currently classified into the following four major molecular subgroups: wingless (WNT)-activated, Sonic hedgehog (SHH)-activated, and the non-WNT/non-SHH subgroups known as Group 3 and Group 4 [1,18,19]. Each subgroup is characterized by unique developmental origins, signaling pathways, and clinical outcomes and this molecular stratification has become essential for risk assessment, therapy decisions, and the design of clinical trials [20,21]. More recently, unique subtypes within subgroups have been identified on the basis of marked intra- and intertumoral heterogeneity [22,23,24,25].

WNT-subtype MB, which exhibits a relatively favorable prognosis, typically arises from mutational activation of the WNT signaling pathway in neuronal precursors in the lower rhombic lip (RL) and harbors mutations in exon 3 of catenin β1 (*CTNNB1*) and monosomy of chromosome 6 [13,26]. The RL is a specialized region within the embryonic hindbrain where proliferating neural precursor cells are generated. The lower RL serves as the germinal zone for mossy fiber and climbing fiber neurons in the brainstem nuclei, whereas the upper RL constitutes the primary source of cerebellar granule cells (CGCs), the most abundant type of neuron in the cerebellum. The upper RL also gives rise to GABAergic cerebellar nucleus neurons, Purkinje cells, interneurons, glutamatergic cerebellar nuclei, and unipolar brush cells [27,28]. Mutations that activate the SHH pathway originate SHH-subtype MB in granule cell precursors (GCPs, also called granule neuron precursors or granule neuron progenitors (GNPs)) from the upper RL [29,30,31,32]. In SHH MB, patched (*PTCH*), smoothened, frizzled-class receptor (*SMO*), and suppressor of fused homolog (*SUFU)* mutations are common, and the tumor protein 53 (*TP53*) mutation status plays a critical role, with *TP53*-mutant tumors being associated with the higher risk and frequent amplification of the MYCN proto-oncogene, BHLH transcription factor (*MYCN*). A poor prognosis is also indicated with *MYCN* amplification by itself in SHH MB, regardless of metastatic dissemination [33,34,35]. Group 3 and Group 4 MB are clinically heterogenous and particularly aggressive, accounting for a significant proportion of metastatic and relapsed cases. These subgroups likely have a common origin in glutamatergic cells within the fetal RL, suggesting that they share a spectrum of developmentally linked disease types. This possible unified origin is consistent with the overlap in biological and clinical features and the anatomical location of these two subgroups [36]. The origin of Group 3 MB has been traced to a population of stem-like cells expressing protogenin (PRTG) in the RL ventricular zone [37]. A recent study showed that overexpression of the development transcriptional regulator Zic family member 1 (ZIC1) inhibits the growth of Group 3 MB whilst promoting the proliferation of SHH MB precursor cells, highlighting the crucial importance of biological context within different subgroups [38]. Further complicating MB biology is the presence of tumor-initiating or cancer stem cells (CSCs), a subpopulation capable of self-renewal and multilineage differentiation. These cells, marked by proteins such as prominin-1 (CD133), SRY-box transcription factor 2 (SOX2), and polycomb complex protein BMI-1 (BMI-1), contribute to therapy resistance and tumor recurrence. Experimental models have shown that CD133+ MB cells can form neurospheres in vitro and recreate tumors in immunocompromised mice, reinforcing their role in MB pathogenesis [39,40,41,42,43,44]. PRTG may be a driver of CSCs involved in the initiation and progression of Group 3 MB [37,40].

Together, this evidence indicates that, as with other childhood cancers, MB is a developmental disease and its origins can be traced back to discrete errors in embryogenesis affecting specific neural cell precursor types involved in cerebellar formation. Understanding the cellular origins and molecular basis of MB is critical to allow targeting of the specific pathways and progenitor populations involved in each MB subtype, advancing the discovery of more effective and less toxic personalized therapeutic strategies.

## 3. The GABAergic System and Its Role in Inhibitory Neurotransmission, Brain Development, and Synaptic Plasticity

**Figure 1:**
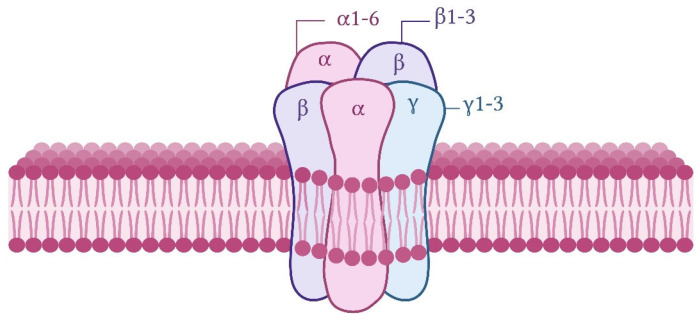
Schematic illustration of a typical subunit composition of a functional GABA_A_ receptor in the CNS. The receptor is a hetero-pentameric GABA-gated Cl^−^ channel composed of a combination of five subunits.

**Figure 2:**
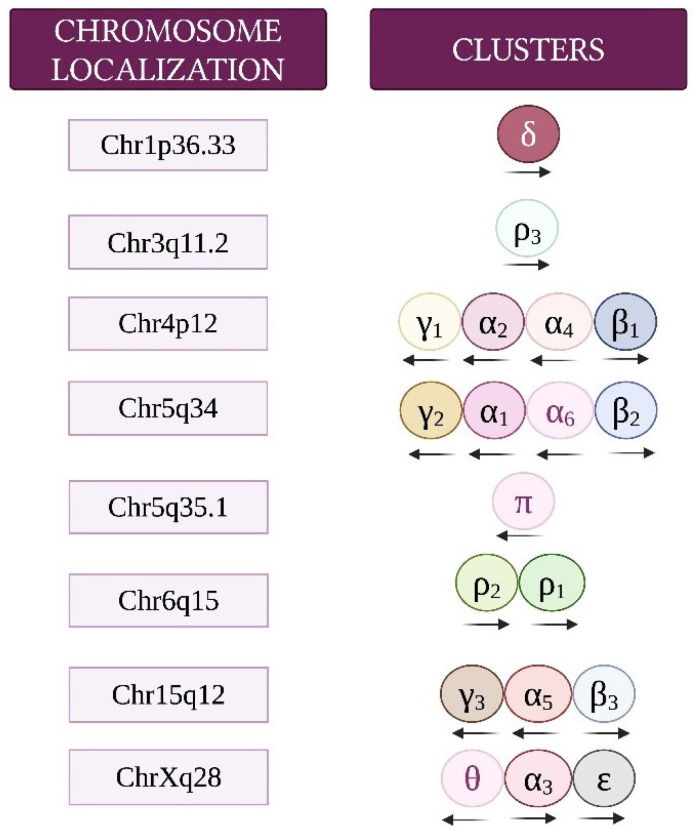
Chromosome locations and cluster organization of genes encoding GABA_A_ receptor subunits. Transcriptional orientation is indicated by arrows. Adapted from [48].

GABA, mainly released from GABAergic inhibitory interneurons, acts by activating the GABA_A_, GABA_B_, and GABA_C_ types of receptors. GABA_A_ and GABA_C_ receptors are ionotropic and associated with Cl^−^ channels, generally allowing neuronal hyperpolarization and fast inhibitory neurotransmission. These two types of receptors can be pharmacologically distinguished on the basis of their differential sensitivities to inhibitors. GABA_A_ receptors are inhibited by bicuculline, whereas GABA_C_ receptors are sensitive to (1,2,5,6-tetrahydropyridin-4-yl) methylphosphinic acid (TPMPA). GABA_B_ receptors are metabotropic and act through G protein (primarily Gi/o) stimulation, leading to the activation of G protein-gated inward-rectifier potassium channels and inhibition of voltage-gated calcium channels and adenylyl cyclase. These effects result in a slower neuronal inhibitory action [2,3,45,46,47]. GABA_A_ and GABA_C_ receptors are members of the Cys-loop superfamily of ligand-gated receptors made up of pentameric protein subunits. This review mostly focuses on GABA_A_ receptors, which, at the postsynaptic membrane, mediate most inhibitory neurotransmissions throughout the adult brain and are the ones shown to possibly influence MB. In addition to the GABA binding site, GABA_A_ receptors have several modulatory binding sites, including sites activated by benzodiazepines, barbiturates, ethanol, neurosteroids, and anesthetics, including propofol [2]. Structurally, GABA_A_ receptors consist of a combination of five proteins drawn from a repertoire of 19 subunits (α1–6, β1–3, γ1–3, δ, ε, θ, π, and ρ1–3). Most functional GABA_A_ receptors consist of two α, two β, and one γ or δ subunit/s (Figure 1). Of the 19 genes encoding GABA_A_ receptor subunits, 14 are organized in clusters on human chromosomes 4, 5, and 15, as well as chromosome X, and each cluster contains genes coding for α, β, or γ/ε subunits. *GABRD* (δ subunit) and *GABRR3* (ρ3 subunit) are orphans on chromosome 1 and 3, respectively. *GABRP* (π subunit) is located on chromosome 5 and is separate from the γ2α1α6β2 gene cluster. *GABRR1* (ρ1 subunit) and *GABRR2* (ρ2 subunit) are located on chromosome 6 (Figure 2) [48,49,50].

A finely regulated balance between neuronal excitation and inhibition (mediated mostly by glutamatergic and GABAergic synapses, respectively) is required for normal CNS functioning and synaptic plasticity. GABA_A_ activation plays an overall inhibitory role in neural plasticity processes such as long-term potentiation (LTP) at excitatory synapses [51,52]. However, GABA_A_ receptor desensitization facilitates LTP induction at inhibitory synapses in the hippocampus and cortical areas, thus contributing to plasticity related to long-term activity-dependent changes in inhibitory efficacy [53,54]. In contrast to its predominant role in the adult CNS, GABA_A_ activation mediates GABA-induced neuronal excitation during early neural development, an effect that is at least partially caused by altered Cl^−^ gradients. Functional GABAergic neurons develop earlier than glutamatergic neurons during embryogenesis, and GABA may provide the main excitatory drive during early neurodevelopment [55,56,57,58,59]. GABA-evoked inward currents could be detected in neurons cultured from E15 embryos [60]. Interneurons are probably the first neurons to generate network-driven activity in the developing hippocampus, inducing GABA-dependent postsynaptic potentials that play an important role in enhancing synaptic efficiency between excitatory neurons [61]. The requirement of GABA_A_ receptor activity for the normal development of hippocampal and cortical structures and connectivity has been shown in several experimental models [61,62].

## 4. GABAergic Regulation of Cerebellar Development and Function: Focus on GPCs and CGCs

The cerebellum is derived from the dorsal part of the anterior hindbrain and contains two groups of neurons, glutamatergic CGCs and GABAergic Purkinje cells. CGCs derive from GCPs in the RL, whereas Purkinje cells derive from progenitors in the ventricular zone. Both cerebellar neuronal types receive input from mossy and climbing fibers external to the cerebellum [63]. The cerebellum shows a high level of neurogenesis after birth, although cerebellar neurogenesis does not persist into adulthood. Neurons generated early after birth become CGCs, which are glutamatergic interneurons that outnumber all other neuronal types in the CNS combined. As noted above, MB can originate from GCPs, which give rise to CGCs. Beginning at embryonic day 10 in mice, GCPs migrate from the RL to originate the external germinal layer (EGL) [64,65,66]. GCPs in the EGL express ionotropic glutamate receptors [67,68] and also functional GABA_A_ receptors [69]. Messenger RNAs for the α2 and α3 subunits are not observed in proliferating GCPs in the EGL, but appear in the differentiating zone of the developing cerebellum at embryonic day 13 (E13), and the α2 subunit is detected in migrating and differentiating CGCs until postnatal day 14 (PN14). α3 subunit mRNA is found in developing Purkinje cells and cerebellar nucleus neurons, disappearing from Purkinje cells by the end of the PN week. It is possible that GABA extrasynaptically activates α2- and α3-subunit-containing GABA_A_ receptors on differentiating neurons, contributing to differentiation prior to the formation of mature synapses and networks in the cerebellum [70]. In addition to α2 and α3 subunits, protein expression of the α1 GABA_A_ receptor subunit can also be detected in GCPs [71,72]. GABA_A_ receptor α1 and γ2 subunits, but not the α6 or δ subunits, form clusters in CGC neurites, irrespective of the presence of GABAergic axons [73]. Both the GABAB1 and GABAB2 subunits of the GABA_B_ receptor show overlapping distributions in the cerebellar cortex, both at pre- and postsynaptic sites, during development, particularly in Purkinje cells. These GABA_B_ receptors may play a role in the maturation of excitatory glutamatergic synapses [74,75]. The binding of [3H]GABA to GABA_B_ receptors in the cerebellar molecular layer peaks between PN 14 and PN 28 and then decreases to adult levels. Transient high expression of GABA_B_ receptors occurs in the deep cerebellar nuclei, peaking at PN 3 and decreasing to adult levels by PN 21 [76]. All three ρ subunits of the GABA_C_ receptor are expressed during cerebellar development, particularly in the soma and dendritic tree of Purkinje cells [77].

Exposure to GABA or the selective GABA_A_ receptor agonist muscimol induces depolarization and an increase in Ca^2+^ levels in GPCs in parasagittal cerebellar slices from PN 8 mice. It remains to be fully clarified what endogenous sources of GABA could excite GPCs in the EGL [69], but CGCs can be inhibited by GABA being synaptically released from Golgi cells, as well as by the tonic activity of α6-subunit-containing GABA_A_ receptors [78,79,80]. Many other studies show that developing or mature CGCs are responsive to GABA. For instance, both GABA and muscimol stimulate the proliferation of immature CGCs, an effect that is blocked by either the GABA_A_ receptor picrotoxin or mitogen-activated protein kinase (MAPK) inhibition [81]. Exposure to GABA or GABA_A_ receptor agonists can also promote the morphological differentiation of developing CGCs, as evidenced by the stimulation of neurite formation and an increase in the cytoplasmic density of organelles involved in protein synthesis and processing [82,83,84]. Stimulation of the benzodiazepine binding site on GABA_A_ receptors by diazepam, clobazam, and RL 214 leads to responses in receptors containing α1, β2, and γ2 subunits [85]. The sensitivity to GABA increases in recordings made from days 7 to 11 in cultured CGCs, and furosemide, which inhibits α6-containing GABA_A_ receptors, impairs GABA-induced currents more potently from days 11 to 14 than at day 7 [86]. Inhibitory postsynaptic currents change during CGC development, likely due to regulation of the presynaptic uniquantal release of GABA [87]. The expression of GABA_A_ receptor subunits in CGCs during development is regulated by neuronal depolarization [88,89], protein tyrosine kinases, and protein kinase C (PKC) [90], and the activity of extrasynaptic GABA_A_ receptors in CGCs is modulated by tonically active GABA_B_ receptors, adenylate cyclase, protein kinase A (PKA), calcium/calmodulin-dependent protein kinase II (CaMKII), and the release of Ca^2+^ from intracellular stores [91]. GABA_B_ receptors mediate G-protein-independent inward-rectifier K^+^ currents in the CGCs of PN 19–26 rats [92].

This evidence strongly indicates that although cerebellar CGCs are glutamatergic, (1) these cells express GABA_A_, GABA_B_, and GABA_C_ receptors containing multiple subunit combinations; (2) GABA_A_ and GABA_B_ receptors in developing or mature CGCs are functional and can display tonic activity; and (3) GABA receptor activity can significantly influence CGC differentiation and integration into cerebellar synaptic circuits.

## 5. The GABAergic System and Cancer

GABA is found in different types of solid tumors including breast, gastric, ovarian cancer, and glioma [93,94]. In addition to acting as a neurotransmitter, GABA is also a metabolite in the Krebs cycle and is, therefore, found in non-neuronal cells, including cancer cells, where it can influence proliferation, differentiation, and migration [95,96]. A study based on samples from patients with lung squamous cell carcinoma, lung adenocarcinoma, and colon adenocarcinoma patients from The Cancer Genome Atlas (TCGA) reported that GABA content is associated with advanced stages of cancer, and high levels of GABA predict mortality in patients with lung and colon cancer [97]. In addition, the levels of GABA-producing enzyme glutamate decarboxylase 1 (GAD1) are increased in tumor samples specifically from these patients. A high intra-tumoral GAD1 expression is associated with a poorer prognosis, whereas the expression of GABA transaminase (4-aminobutyrate aminotransferase, or ABAT), which metabolizes GABA, is reduced in lung squamous cell carcinoma and colon adenocarcinoma. Furthermore, the findings show that GABA stimulates GABA_B_ receptors to enhance β-catenin signaling through a mechanism dependent on the inhibition of glycogen synthase kinase 3 (GSK-3), resulting in increased cancer cell proliferation and the suppression of CD8-positive T cell infiltration in tumors [93,97].

In a cohort of 89 breast cancer patients, overall survival significantly increased in patients with a high level of GABA measured in tumor homogenates in comparison to patients with low GABA levels. The median overall survival was 127.2 months in patients with a high GABA level (>89.3 μg/1) compared with 106.4 months in the group with a low level [98]. The GABA_A_ receptor β3 subunit is expressed at higher levels in triple-negative breast cancer cell lines compared with non-tumor MCF10A cells, and its knockdown results in reduced proliferation and migration, in addition to cell cycle arrest associated with decreased cyclin D1 and increased p21 expression [99], as well as a reduction in GABA_A_ receptor-mediated Cl^−^ cellular influx [100]. Expression of the π subunit of the GABA_A_ receptor is associated with basal-like and triple-negative types of breast cancer and correlates with poor patient outcomes [101].

The π subunit is also overexpressed in pancreatic ductal adenocarcinoma (PDAC), and knockdown of *GABRP*, the gene that encodes the π subunit, inhibits PDAC cell growth. The addition of GABA to a cell culture medium selectively promotes the proliferation of *GABRP*-expressing cells, increases intracellular Ca^2+^ levels, and stimulates the MAPK signaling pathway [102]. The knockdown of GAD1 expression inhibits proliferation, migration, and invasion in oral squamous cell carcinoma cells by reducing the expression of GABA_B_ receptors, and the effects are reversed by exposure to GABA [103].

In advanced prostate cancer, the GABA content can be increased by phosphorylation and activation of GAD1, and GABA regulates nuclear androgen receptor signaling to promote tumorigenesis [104]. Prostate cancer neuroendocrine cells show increased activity of the GABAergic system, including enhanced expression of the GABBR1 subunit of GABA_B_ receptors, which may contribute to tumor progression [105]. Patient-derived castration-resistant prostate tumor xenografts show increased GABAergic activation due to phosphorylation and activation of glutamate decarboxylase 65 (GAD65), which synthesizes GABA from glutamate by decarboxylation. In these tumors, GABA binds to and retains the androgen receptor through associations with the nuclear zinc finger protein ZNHIT3. The knockdown of GAD65 reduces the growth of prostate tumor xenografts and delays the emergence of castration resistance [106].

The GABAergic system is also involved in the metastatic capacity of peripheral solid tumors. Glutamic pyruvate transaminase (GPT2) catalyzes the transamination between alanine and α-ketoglutarate (α-KG) to generate pyruvate and glutamate, which can be converted to GABA. The overexpression of GPT2 results in an increase in GABA content and the GABA_A_-receptor-mediated promotion of metastasis in experimental breast cancer. The δ receptor subunit GABRD is required for GPT2/GABA-induced metastasis; *Gpt2* knockout results in reduced lung metastasis of *Gpt2^−/−^* breast cancer and prolongs survival in mice. GABA_A_ receptor activation in GPT2-overexpressing breast cancer causes an increase in Ca^2+^ influx and stimulation of PKC signaling, ultimately activating the transcription factor cAMP response element-binding protein (CREB), which increases the expression of genes related to metastasis, including *PODXL*, *MMP3*, and *MMP9* [107]. Other studies support a role for the GABAergic system in promoting the metastasis of peripheral solid tumors. GABA receptors are expressed in colon cancer cells, and treatment with pentobarbital, which potentiates GABA_A_ activity by binding to the barbiturate binding site, blocks the formation of metastasis from primary colon tumors in mice [108]. GABA levels are markedly increased in both the cells and serum of patients with non-small cell lung cancer (NSCLC) with brain metastases, and GABA enhances the brain metastatic capability of NSCLC cells. The metastasis-capable cells inhibit ABAT through the downregulation of the transcription factor forkhead box A2 (FOXA2), resulting in an accumulation of GABA, which, in turn, activates the nuclear factor kappa B (NF-κB) pathway to promote brain metastasis [109].

In brain tumors, cells from primary cultures of lower-grade gliomas, namely, oligodendroglioma and astrocytoma cells, can either depolarize or hyperpolarize in response to GABA through GABA_A_ receptor activation and increases in cellular Ca^2+^ [110]. GABA metabolism regulates glioblastoma (GBM) stem cell proliferation through the increased formation of the GABA by-product 4-hydroxybutyrate (GHB) [111]. The presence of GABA_A_ receptors in GBM cells is generally associated with less malignant tumors, and receptor expression can be triggered by contact with neurons, highlighting the importance of neuron–tumor interactions in brain tumors [112]. GBM-derived U3047MG cells express mRNA for α2, α3, α5, β1, β2, β3, δ, γ3, π, and θ GABA_A_ receptor subunits, and drugs that potentiate GABA_A_ activity by binding to modulatory binding sites—namely, diazepam, propofol, and etomidate—potentiate GABA-evoked GABA_A_ currents in these cells [113]. A quantitative real-time reverse transcription polymerase chain reaction (qRT-PCR) analysis of the mRNA expression of all 19 GABA_A_ subunits in 29 human glioma samples and 5 peritumoral tissue samples found lower levels in GBM compared with lower-grade gliomas, except for the θ subunit. All subunits, except for the ρ1 and ρ3 subunits, were detected. An immunohistochemical analysis of the expression and distribution of the α1, γ1, ρ2, and θ subunits in tissue microarrays containing 87 grade II glioma tumors found the co-expression of ρ2 and θ subunits in astrocytoma and oligodendroglial tumors. The expression of the ρ2 subunit is associated with longer survival in astrocytoma patients [114]. More recently, the expression of genes encoding the GABA_A_ receptor subunits was characterized for different glioma types in a study using data from the French and The Cancer Genome Atlas Brain Lower Grade Glioma (TCGA-LGG) datasets. The expression of *GABRA2*, *GABRA3*, *GABRB3*, *GABRG1*, and *GABRG2* significantly correlates with patient prognosis assessed by overall survival, with a higher gene expression indicating longer survival for most genes. In patients with GBM, a high expression of *GABRA2* is associated with poorer survival, whereas high *GABRB3* levels are associated with a better prognosis. In patients with lower-grade gliomas, *GABRA3*, *GABRB3*, *GABRG1*, and *GABRG2* correlate with longer survival [115]. A weighted gene co-expression network analysis (WGCNA) of RNAseq data to identify ion channel gene hubs in diffuse midline glioma and GBM found *GABRA1* and *GABRG2* to be targets. In additional experiments, patient-derived GBM explant organoids were found to express *GABRA5*, which encodes the α5 subunit, and the selective α5-containing GABA_A_ receptor antagonist S44819 markedly reduced organoid invasion, whereas the partial GABA_A_ antagonist GABA_A_-compound 1b impaired both proliferation and invasion [116].

These studies exemplify how GABA and GABA receptors, particularly the GABA_A_ type, can significantly modulate cancer cell function and tumor progression, and possibly influence patient prognosis, in several types of peripheral solid tumors as well as adult brain tumors. The tumoral levels of GABA or GAD1 expression have been associated with prognosis in lung, colorectal, and breast cancers. In gliomas, the expression of specific GABA_A_ receptor subunits has been associated with patient survival. Below, we review the evidence suggesting a role for the GABAergic system in pediatric brain cancers, specifically MB.

## 6. GABA Receptor Influences on MB

MHH-MED-3 MB cells share electrophysiological properties with GCPs, displaying K^+^ and Ca^2+^ currents as well as GABA_A_-receptor-mediated Cl^−^ currents in response to GABA exposure [117]. Increased *GABRA5* has been reported in Group 3 MB, and the selective pharmacological activation of α5-containing GABA_A_ receptors with the agonist QHii066 reduces the survival of *GABRA5*-expressing Group 3 MB cells [10]. Benzodiazepine-related compounds designed to preferentially bind to α5-GABA_A_ receptors impair Group 3 MB cell viability by enhancing Cl^−^ efflux. The underlying mechanism may include mitochondrial membrane depolarization, *TP53* upregulation, the cytoplasmic localization of constitutively phosphorylated p53 protein, and Bcl-2-mediated apoptosis [9].

**Figure 3:**
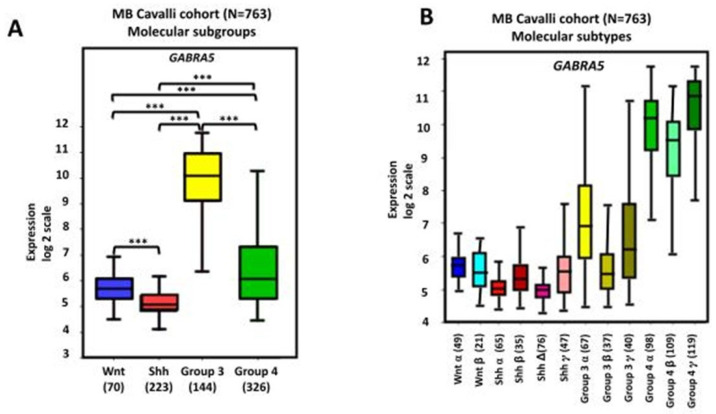
*GABRA5* gene expression in human MB. Data were obtained from the transcriptome dataset comprising 763 tumor samples from patients with MB as previously described by Cavalli et al. (GEO: GSE85218) [22] and analyzed with the R2 Genomics Analysis and Visualization Platform (http://r2.amc.nl). Results are presented in boxplot format as log2-transformed signal intensity. Bars show data for different (**A**) subgroups and (**B**) subtypes of MB; *** *p* < 0.001 compared with other groups, as indicated in the graphs.

**Figure 4:**
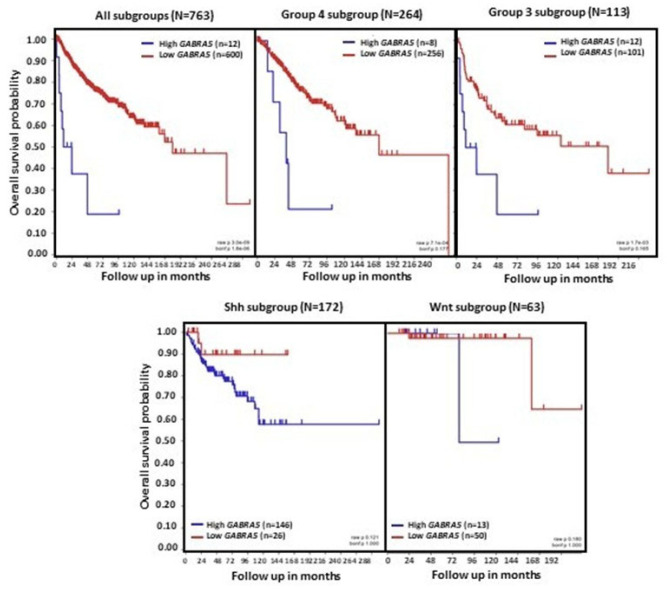
*GABRA5* expression and overall survival in patients with MB. Data were obtained from the transcriptome dataset comprising 763 tumor samples from patients with MB as previously described by Cavalli et al. (GEO: GSE85218) [22]. Data for different molecular subgroups of MB combined or analyzed separately are shown. Patient overall survival was measured from the day of diagnosis until death or date of last follow-up, and calculated using the Kaplan–Meier estimate, with median values and logrank statistics; *p*-values indicated in the panels show significant associations between gene expression and survival in Group 3 and Group 4, but not SHH and WNT MB.

Gene expression analyses using three non-overlapping MB cohorts [18,118,119] showed high levels of *GABRA5* expression in Group 3 MB tumors. A further investigation of α-subunit-containing GABA_A_ receptor expression by immunofluorescence staining found a marked expression in Group 3, but not SHH MB. The expression of α5-GABA_A_ receptors was maintained in both flank and intracranial patient-derived xenografts [10]. An analysis of tumors from the dataset described by Cavalli et al., which contains data from 763 tumors [22], also found a higher expression of *GABRA5* in Group 3 MB, with the highest in the Group 3γ subtype, which has the worst prognosis. In addition, there is a significant positive correlation between *GABRA5* and *MYC* expression in Group 3α MB [9]. A further investigation using this cohort showed high mRNA *GABRA5* expression levels across all four MB subgroups, confirming higher levels in Group 3 MB, particularly in the α, β, and γ subtypes (Figure 3). Importantly, this analysis extends previous studies by showing that high levels of *GABRA5* are associated with a reduction in patient overall survival when all subgroups are pooled together. Detailing the results by an examination of the data separately for each molecular subgroup revealed that a high *GABRA5* expression is related to shorter survival, specifically in patients with Group 3 and Group 4 MB, but not in those with SHH and WNT tumors (Figure 4).

Another study using the Cavalli dataset [22] recently reported that a high *GABRB1* gene expression is associated with better OS within each of the four molecular subgroups. The *GABRB2* gene shows higher transcript levels in Group 3 MB than all other three subgroups, and a high expression is associated with a better prognosis, as measured by overall survival in patients with Group 3 tumors. *GABRB3* expression is significantly higher in Group 3 and Group 4 MB, and a high expression of *GABRB3* is associated with longer survival in patients with SHH tumors. These findings show that high expressions of *GABRB1*, *GABRB2*, and *GABRB3* may be related to a better prognosis in a molecular-subgroup-specific manner, and suggest a role for GABA_A_ receptors containing β subunits in MB [11].

## 7. Concluding Remarks

In this review, we have summarized selected studies that strongly indicate a role for GABA and its receptors in modulating cancer cell function and tumor progression across several types of solid tumors, including brain cancers. This body of evidence highlights neurotransmitter systems within tumor cells or in the tumor microenvironment as regulators of cancer cells and their interactions with surrounding non-tumoral tissue [120,121], and supports the view that membrane receptors should be investigated as potential biomarkers and therapeutic targets, even in the absence of specific activating genetic alterations.

Although current evidence about the involvement of the GABAergic system in MB is still limited, and the relationship between GABA receptor activity, MB cells of origin, and MB development and progression remains unclear, the studies reviewed above indicate that the activity of GABA receptors, particularly GABA_A_ receptors containing the α5 subunit encoded by the *GABRA5* gene, affects MB cells from Group 3 tumors. In terms of potential clinical implications, these findings suggest that drugs that selectively stimulate α5-GABA_A_ receptors can be further explored as potential adjuvant treatments [9,10].

Despite this apparent antitumoral role of α5-GABA_A_ receptors in Group 3 MB, as revealed by cellular pharmacology experiments, a high *GABRA5* expression correlates with a poorer prognosis in patients with Group 3 and Group 4 MB tumors. In addition, GABA_A_ receptor β subunits are differentially associated with patient survival in distinct MB subgroups, suggesting a possible role of these subunits as biomarkers. Future research should extend this evidence by examining the role of GABA_A_, GABA_B_, and GABA_C_ receptors containing distinct subunit repertoires in experimental MB models, as well as possible additional associations between genes encoding GABA receptor subunits and patient prognosis.

In addition to investigating GABA and other neurotransmitters and their receptors within cancer cells, researchers should look at the context of neural–tumor interactions [122,123]. Functional, tumor-promoting synapses between GABAergic neurons and glioma cells mediated by GABA_A_ receptors have been recently described in diffuse midline gliomas (DMGs). GABAergic input depolarizes DMG cells though a mechanism involving NKCC1 Cl^−^ transporter-mediated increases in intracellular Cl^−^ levels. This GABAergic activity, which is enhanced by the benzodiazepine lorazepam, promotes DMG cell proliferation and leads to shorter survival in mouse models. These findings reveal GABAergic synaptic communication between neurons and brain tumors to promote cancer growth [124]. Investigating the MB microenvironment, the structural and functional interactions between MB tumors and surrounding non-tumor neural tissues, and the role of GABAergic and other neurotransmitter systems in mediating or regulating these interactions may represent novel research avenues in the field of pediatric brain cancer biology.

## Data Availability

The dataset analyzed in this study is available in the Gene Expression Omnibus repository, https://www.ncbi.nlm.nih.gov/geo/query/acc.cgi?acc=GSE85217 (accessed on 20 November 2024).
